# Self-perceived quality of life predicts mortality risk better than a multi-biomarker panel, but the combination of both does best

**DOI:** 10.1186/1471-2288-11-103

**Published:** 2011-07-12

**Authors:** Robin Haring, You-Shan Feng, Jörn Moock, Henry Völzke, Marcus Dörr, Matthias Nauck, Henri Wallaschofski, Thomas Kohlmann

**Affiliations:** 1 Institute for Clinical Chemistry and Laboratory Medicine, University of Greifswald, (Ferdinand-Sauerbruch Str.), Greifswald, (17475), Germany; 2 Institute for Community Medicine, University of Greifswald, (Walther Rathenau Str. 48), Greifswald, (17487), Greifswald, Germany; 3 Department of Cardiology; University of Greifswald, (Friedrich-Loefflerstraße 23a), Greifswald, (17489), Greifswald, Germany

**Keywords:** Health-related quality of life, multiple biomarker panel, all-cause mortality, SF-12, population-based cohort

## Abstract

**Background:**

Associations between measures of subjective health and mortality risk have previously been shown. We assessed the impact and comparative predictive performance of a multi-biomarker panel on this association.

**Methods:**

Data from 4,261 individuals aged 20-79 years recruited for the population-based Study of Health in Pomerania was used. During an average 9.7 year follow-up, 456 deaths (10.7%) occurred. Subjective health was assessed by SF-12 derived physical (PCS-12) and mental component summaries (MCS-12), and a single-item self-rated health (SRH) question. We implemented Cox proportional-hazards regression models to investigate the association of subjective health with mortality and to assess the impact of a combination of 10 biomarkers on this association. Variable selection procedures were used to identify a parsimonious set of subjective health measures and biomarkers, whose predictive ability was compared using receiver operating characteristic (ROC) curves, C-statistics, and reclassification methods.

**Results:**

In age- and gender-adjusted Cox models, poor SRH (hazard ratio (HR), 2.07; 95% CI, 1.34-3.20) and low PCS-12 scores (lowest vs. highest quartile: HR, 1.75; 95% CI, 1.31-2.33) were significantly associated with increased risk of all-cause mortality; an association independent of various covariates and biomarkers. Furthermore, selected subjective health measures yielded a significantly higher C-statistic (0.883) compared to the selected biomarker panel (0.872), whereas a combined assessment showed the highest C-statistic (0.887) with a highly significant integrated discrimination improvement of 1.5% (p < 0.01).

**Conclusion:**

Adding biomarker information did not affect the association of subjective health measures with mortality, but significantly improved risk stratification. Thus, a combined assessment of self-reported subjective health and measured biomarkers may be useful to identify high-risk individuals for intensified monitoring.

## Background

Subjective health measures are widely used within clinical and epidemiological research, as well as health policy settings, being easily assessed by a single-item self-rated health (SRH) question or more thoroughly using health related quality of life (HRQoL) instruments. The Short Form Health Survey (SF-36) is a well documented and validated HRQoL instrument [1-3], with the SF-12 developed as a shorter alternative. With the advantage of two summary scores of physical (PCS-12) and mental component summaries (MCS-12), the SF-12 has been extensively applied in epidemiological studies [4].

In particular, the relationship between SRH and mortality has been repeatedly reported [5-8], suggesting a single measure of SRH as strong predictor of poor overall health status and increased mortality risk [9]. However, previous studies are limited by the fact that the associations between subjective health and mortality have been assessed in elderly [10-12] or disease-specific patient populations including conditions such as cancer [13,14], diabetes mellitus [15], coronary artery disease [16,17], respiratory disease [18,19], chronic kidney disease [20], or infection by HIV [21]. Beside these limitations, the impact and comparative predictive performance of different biomarkers on the association between subjective health measures and mortality risk is largely unknown. This is even more intriguing, as the multi-biomarker approach has recently gained widespread attention as powerful predictors of clinical [22,23] and subclinical outcomes [24].

The present study aims to investigate the impact and comparative predictive performance of a multi-biomarker panel on the association of subjective health with mortality, analyzing data from the 10-year follow-up of the population-based Study of Health in Pomerania (SHIP).

## Methods

### Study population

The SHIP is a population-based cohort study in West Pomerania, conducted in the north-eastern area of Germany comprising the cities of Greifswald, Stralsund, Anklam and 29 surrounding communities with a total of 212,157 residents [25,26]. A representative sample of 7,008 adults aged 20 to 79 years was invited to participate. A two-stage cluster sampling method was adopted for this purpose from the WHO MONICA in Germany (Augsburg) and yielded twelve five-year age strata for both genders, each including 292 individuals in a total of 34 towns or villages. Only individuals with German citizenship and main residency in the study area were included. The net sample (without migrated or deceased persons) comprised 6,267 eligible subjects, of which 4,308 finally participated (response proportion 68.8%). Data collection was performed in two examination centers (Greifswald and Stralsund) between October 1997 and May 2001 after written consent was obtained from each participant. The study conformed to the ethical guidelines of the Declaration of Helsinki as reflected in an a priori approval by the local Ethics Committee of the University of Greifswald. Subjects with missing data for the modelled variables (N = 49) were excluded, yielding a study population of 4,259 individuals.

### Measures

A computer-assisted personal interview assessed socio-demographic information including age, gender, educational level (< 10, = 10, or > 10 years of education), civil status (cohabitation), and occupational status (having no paid job, worker, employed, academic/self-employed); health-related behaviour including physical activity (physical training during summers or winters for at least one hour a week), excessive/high-risk alcohol consumption (> 30 g alcohol/day for men and > 20 g alcohol/day for women [27]), smoking habits (current, former, or never-smoker), and diet (gender-specific tertiles from a validated food-frequency questionnaire reflecting food quality [28]); as well as subject's self-reported medical history including hypertension, myocardial infarction, stroke, and diabetes mellitus. Because income is a household-level variable, "equalized" household income (in Euros) was calculated using the commonly adopted procedure of the Luxembourg Income Study to divide the household income by the square root of the number of household members [29]. Somatometric measures included waist circumference (WC), measured to the nearest 0.1 cm using an inelastic tape midway between the lower rib margin and the iliac crest in the horizontal plane with the subject standing comfortably with weight distributed evenly on both feet. We measured HRQoL using the two SF-12 components PCS-12 and MCS-12. To assess SRH, we used the single-item question: "Over the last 12 months would you say your health has been very good, good, fair, poor, or very poor?"

Laboratory assessment of the multi-biomarker panel included measurement of 10 biomarkers from distinct biological pathways associated with increased morbidity and mortality including inflammation [high sensitive C-reactive protein (hs-CRP)], hemostasis (fibrinogen), metabolic disturbances [glycated hemoglobin (HbA1c), total cholesterol, and triglycerides], liver disease [gamma glutamyltransferase (GGT)], kidney disease [urine albumin and glomerular filtration rate (GFR)], thyroid status [thyrotropin (TSH)], and hypothalamic-pituitary-adrenal axis activity [insulin-like growth factor-I (IGF-I)]. Biomarkers were measured as follows: hs-CRP determined immunologically on a Behring Nephelometer II with commercially available reagents from Dade Behring (Dade Behring, Eschborn, Germany); plasma fibrinogen assayed according to Clauss using an Electra 1600 analyzer (Instrumentation Laboratory, Barcelona, Spain); HbA1c determined by high-performance liquid chromatography (Bio-Rad Diamat, Munich, Germany); total cholesterol measured photometrically (Hitachi 704, Roche, Mannheim, Germany); triglyceride determined enzymatically using reagents from Roche Diagnostics (Hitachi 717, Roche Diagnostics, Mannheim, Germany); urine albumin determined on a Behring Nephelometer (Siemens BN albumin; Siemens Healthcare, Marburg, Germany); GGT measured photometrically (Hitachi 717; Roche Diagnostics, Mannheim, Germany); creatinine determined with the Jaffé method (Hitachi 717, Roche Diagnostics, Germany) and the GFR estimated according to the modified MDRD formula [30]; TSH measured by immunochemiluminescent procedures (Byk Sangtec Diagnostica, Frankfurt, Germany); and IGF-I determined by automated two-site chemiluminescence immunoassays (Nichols Advantage; Nichols Institute Diagnostica GmbH, Bad Vilbel, Germany) [26].

Information on vital status were acquired at regular intervals from time of enrollment into the study through December 15, 2009. Individuals were censored at either death or loss to follow-up. The number of months between baseline examination and censoring was used as follow-up length.

### Statistical analysis

Data on quantitative and qualitative characteristics are expressed as median (inter-quartile range), or percent, respectively. Intergroup comparisons with regard to vital status were performed using χ^2 ^test (qualitative data) or a Mann-Whitney-U test (quantitative data). PCS-12 and MCS-12 were divided into quartiles to calculate crude incidence rates (per 1000 person-years) and to perform multivariable Cox proportional-hazards regression models associating PCS-12, MCS-12, and SRH with all-cause mortality. Kaplan-Meier survival curves were graphed for SRH and compared using the log-rank test. First, we prespecified gender- and age-adjusted Cox models and included alternately socio-demographic factors (civil status, educational level, occupational status, and equalized income), behavioral factors (smoking status, alcohol consumption, physical activity, food consumption, and WC), comorbidities (hypertension, myocardial infarction, stroke, and diabetes mellitus), and the multi-biomarker panel (hs-CPR, fibrinogen, HbA1c, total cholesterol, triglyceride, GFR, albumin, GGT, TSH, and IGF-I). In secondary analyses, we used backward (p ≥ 0.20 for removal) and forward elimination (p < 0.05 for inclusion) procedures to identify a parsimonious adjustment set among all of the applied standard covariates and the multi-biomarker panel.

To compare the predictive performance of the implemented models, we measured the area under the receiver operating characteristic (ROC) curve, or C-statistic, and tested their differences using STATA's "roccomp" command. The C-statistic ranges from 0.5 (no discrimination) to a theoretical maximum of 1 (perfect discrimination) and is equivalent to the probability that the predicted risk is higher for a case (decedent) than for a non-case (survivor) [31]. Furthermore, we estimated the integrated discrimination improvement (IDI) to examine whether the prediction on the basis of a model without the biomarker panel was significantly improved after inclusion of the biomarker panel [32]. In contrast to the net reclassification improvement which needs a priori meaningful predicted risk categories, the integrated discrimination improvement is based on continuous differences in the predicted risk from new and old models. IDI were obtained with logistic regression models that examined deaths through 10-years of follow-up.

We assessed the potential effect modification of the investigated associations by age and gender through additional inclusion of multiplicative interaction terms (age and gender * HRQoL and SRH, respectively) into the applied multivariable Cox models. To account for the potential impact of changing risk factor patterns over time, we entered the applied adjustment sets as time-varying covariates into the analysis. Further sensitivity analyses were performed by recalculating the applied models stratified by 20-year age-groups and gender, as well as adjusting for possible non-response bias by using inverse probability weights [33]. An elevated level of item nonresponse of > 5% was an issue with regard to the MCS-12 and PCS-12. Therefore we used multiple imputations by chained equations (MICE) as an extremely suitable algorithm to obtain completed versions of incomplete MCS-12 and PCS-12 [34,35]. Item nonresponse from all further variables was less than 2%. We verified that the assumption of proportionality of hazards was satisfied. Hazard ratios (HR) were calculated with a 95% confidence interval (95% CI). We considered two-sided P value less than p < 0.05 to be statistically significant. This manuscript was written in accordance with the STROBE statement, giving guidelines for reporting of observational studies [36]. All statistical analyses were performed using Stata 11.0 (Stata Corporation, College Station, TX).

## Results

Data on subjective health measures, biomarkers, and covariates are presented in Table 1. During 41,180 person-years (mean, 9.7 years; 25^th^, 9.3; 75^th^, 10.7) of follow-up, 456 individuals (10.7%) died, resulting in an overall death rate of 11.1 deaths per 1000 person-years. Crude incidence rates of all-cause mortality decreased across quartiles of PCS-12 but not MCS-12 (Table 2).

**Table 1:** Baseline characteristics of the study population stratified by vital status.

Characteristics	Survivors (N = 3,803)	Decedents (N = 456)	p *
Gender, female	53.2	32.2	< 0.001
Age, years	47.0 (34.2; 60.4)	70.3 (63.4; 76.2)	< 0.001
Physical component scale (PCS-12)	48.1 (44.2; 50.3)	43.9 (39.1; 48.8)	< 0.001
Mental component scale (MCS-12)	44.4 (40.7; 47.6)	46.1 (41.6; 50.2)	< 0.001
General health, %			< 0.001
very good & good	18.7	5.3	
fair	64.5	53.7	
poor & very poor	16.8	41.0	
Civil status (cohabiting), %	76.3	67.4	< 0.001
Educational level, %			< 0.001
< 10 y	35.5	75.3	
= 10 y	48.3	17.6	
> 10 y	16.2	7.1	
Occupational status, %			< 0.001
Worker	15.7	2.9	
Employee	32.4	4.4	
Academic/self-employed	5.9	1.8	
No paid job	46.0	90.9	
Household income, €	949.0 (636.8; 1214.3)	970.5 (703.0; 1175.0)	0.464
Smoking status, %			< 0.001
Never	36.1	32.2	
Former	32.8	42.9	
Current	31.1	24.9	
Subject's medical history, %			
Hypertension	20.8	46.2	< 0.001
Myocardial infarction	2.6	9.6	< 0.001
Stroke	1.6	8.1	< 0.001
Diabetes mellitus	4.8	20.2	< 0.001
Riskful alcohol consumption, %	14.8	12.2	0.137
Physically active, %	44.1	24.5	< 0.001
Waist circumference, cm	88.1 (78.0; 98.2)	97.2 (89.0; 104.2)	< 0.001
Food consumption, %			< 0.001
Unfavorable	35.4	48.1	
Regular	22.5	27.1	
Optimal	42.1	24.8	
High sensitive C-reactive protein, mg/l	1.25 (0.62; 2.90)	2.48 (1.17; 5.59)	< 0.001
Fibrinogen, g/l	2.83 (2.50; 3.33)	3.25 (2.76; 3.80)	< 0.001
Glycated hemoglobin, %	5.3 (4.9; 5,7)	5.8 (5.3; 6,4)	< 0.001
Total cholesterol, mmol/l	5.66 (4.91; 6.47)	5.89 (5.15; 6.55)	0.009
Triglycerides, mmol/l	1.45 (0.99; 2.22)	1.74 (1.24; 2.68)	< 0.001
Albumin (urine), mg/l	7.5 (4.2; 15.2)	12.1 (6.1; 33.6)	< 0.001
Gamma glutamyltransferase, μmol/L s	0.33 (0.23; 0.55)	0.40 (0.27; 0.76)	< 0.001
Glomerular filtration rate, ml/min	75.3 (66.7; 84.1)	66.8 (56.8; 78.4)	< 0.001
Thyrotropin, mU/l	0.67 (0.45; 0.98)	0.59 (0.36; 0.91)	< 0.001
Insulin-like growth factor-I, ng/ml	136.0 (105.2; 174.4)	107.5 (82.6; 142.2)	< 0.001

Data are percentages or median (25^th^; 75^th^).

* p-values based on χ^2 ^(qualitative data) or Mann-Whitney-U test (quantitative data).

**Table 2:** Crude incidence rates of all-cause mortality by quartiles of PCS-12 and MCS-12.

	PCS-12 in quartiles			
	
	1. Quartile	2. Quartile	3. Quartile	4. Quartile
N	1,054	1,065	1,021	1,119
Person-years	9,715	10,416	9,945	11,102
Number of Deaths (crude incidence rate per 1000 person-years)	221 (22.7)	93 (8.9)	81 (8.1)	61 (5.5)
	**MCS-12 in quartiles**			
	
	**1. Quartile**	**2. Quartile**	**3. Quartile**	**4. Quartile**

N	1,070	1,103	1,023	1,063
Person-years	10,458	10,799	9964	9959
Number of Deaths (crude incidence rate per 1000 person-years)	96 (9.2)	90 (8.3)	97 (9.7)	173 (17.4)

PCS-12, Physical Component Summary; MCS-12, Mental Component Summary.

**Figure 1:**
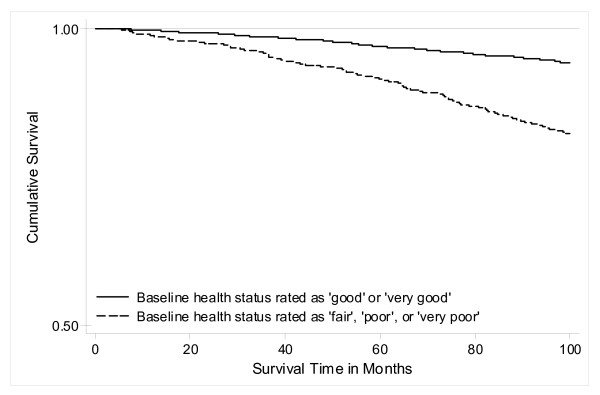
**Kaplan-Meier survival curves for self-rated health associated with 10-year mortality risk**. Subjects with "fair", "poor", or "very poor" self-rated health had a significantly shorter survival compared to subjects with "good" or "very good" self-rated health (log-rank test; p < 0.001).

In Cox proportional-hazards models adjusted for gender and age, we found a distinct association between low PCS-12 scores and all-cause mortality, showing that subjects with PCS-12 scores in the lowest quartile had an increased mortality risk (HR 1.75; 95% CI 1.31-2.33) compared to subjects in the highest quartile. The inclusion of potentially confounding socio-demographic factors (HR, 1.63; 95% CI, 1.22-2.17), behavioral factors (HR, 1.78; 95% CI, 1.33-2.40), comorbidities (HR, 1.60; 95% CI, 1.19-2.14), as well as the multi-biomarker panel (HR, 1.64; 95% CI, 1.19-2.27), attenuated the estimates only slightly (Table 3). P for trend statistics confirmed that HRs were linearly elevated across PCS-12 quartiles in all applied models (p < 0.001). Cox proportional-hazards analyses for low MCS-12 scores did not yield any associations with all-cause mortality (Table 3). We further conducted Cox models for the association of SRH with all-cause mortality and revealed that subjects reporting "poor" or "very poor" SRH had a twofold higher mortality risk (HR 2.07; 95% CI 1.34-3.20) compared to subjects reporting "good" or "very good" SRH. Again, additional adjustment altered the relationship only slightly (Table 4). The complete regression results were given as Additional file 1. Kaplan-Meier survival curves additionally indicated that subjects with "fair", "poor", or "very poor" SRH had significantly (log-rank test: p < 0.001) shorter survival times compared to subjects who reported their SRH as "good" or "very good" (Figure 1).

**Table 3:** Adjusted hazard ratios (HR, 95% CI) for quartiles of PCS-12 and MCS-12 associated with all-cause mortality.

	PCS-12 in quartiles			
	
(Ref.: highest quartile)	min/43.6	43.6/47.5	47.5/50.1	p for Trend
Model 1: adjusted for age & gender	1.75 (1.31; 2.33) *	1.12 (0.81; 1.55)	1.07 (0.77; 1.49)	< 0.001
Model 1 + socio-demographic factors ^a^	1.63 (1.22; 2.17) *	1.08 (0.78; 1.50)	1.03 (0.74; 1.44)	< 0.001
Model 1 + behavioral factors ^b^	1.78 (1.33; 2.40) *	1.13 (0.81; 1.58)	1.08 (0.77; 1.52)	< 0.001
Model 1 + comorbidities ^c^	1.60 (1.19; 2.14) *	1.08 (0.77; 1.49)	1.03 (0.74; 1.45)	< 0.001
Model 1 + biomarker panel ^d^	1.64 (1.19; 2.27) *	1.08 (0.75; 1.55)	1.08 (0.75; 1.56)	< 0.001
	**MCS-12 in quartiles**			
	
	**min/40.8**	**40.8/44.4**	**44.4/48.0**	**p for Trend**

Model 1: adjusted for age & gender	0.97 (0.75; 1.24)	0.91 (0.70; 1.17)	0.84 (0.65; 1.08)	0.759
Model 1 + socio-demographic factors ^a^	0.98 (0.76; 1.26)	0.98 (0.75; 1.26)	0.87 (0.68; 1.12)	0.944
Model 1 + behavioral factors ^b^	1.06 (0.82; 1.37)	0.95 (0.73; 1.23)	0.88 (0.68; 1.13)	0.707
Model 1 + comorbidities ^c^	0.96 (0.74; 1.23)	0.92 (0.71; 1.19)	0.87 (0.68; 1.12)	0.689
Model 1 + biomarker panel ^d^	0.97 (0.74; 1.28)	0.93 (0.70; 1.23)	0.85 (0.65; 1.12)	0.840

* p < 0.05; HR, hazard ratio; 95% CI, 95% confidence interval; PCS-12, Physical Component Summary; MCS-12, Mental Component Summary.

a, HR adjusted for age, gender, civil status, educational level, occupational status, and equalized income.

b, HR adjusted for age, gender, smoking status, alcohol consumption, physical activity, food consumption, and waist circumference.

c, HR adjusted for age, gender, previous history of hypertension, myocardial infarction, stroke, and diabetes mellitus.

d, HR adjusted for age, gender, high sensitive C-reactive protein, fibrinogen, glycated hemoglobin, total cholesterol, triglycerides, glomerular filtration rate, albumin, gamma glutamyltransferase, thyrotropin, and insulin-like growth factor-I.

**Table 4:** Adjusted hazard ratios (HR, 95% CI) for self-rated health (SRH) associated with all-cause mortality.

	SRH in categories	
	
(Ref.: subjects rating SRH "very good" or "good")	"fair"	"poor & very poor"
Model 1: adjusted for age & gender	1.23 (0.81; 1.88)	2.07 (1.34; 3.20) *
Model 1 + socio-demographic factors ^a^	1.17 (0.76; 1.78)	1.88 (1.21; 2.90) *
Model 1 + behavioral factors ^b^	1.16 (0.75; 1.80)	2.00 (1.28; 3.12) *
Model 1 + comorbidities ^c^	1.18 (0.77; 1.81)	1.87 (1.20; 2.91) *
Model 1 + biomarker panel ^d^	1.11 (0.70; 1.75)	1.63 (1.02; 2.62) *

* p < 0.05; HR, hazard ratio; 95% CI, 95% confidence interval.

a, HR adjusted for age, gender, civil status, educational level, occupational status, and equalized income.

b, HR adjusted for age, gender, smoking status, alcohol consumption, physical activity, food consumption, and waist circumference.

c, HR adjusted for age, gender, previous history of hypertension, myocardial infarction, stroke, and diabetes mellitus.

d, HR adjusted for age, gender, high sensitive C-reactive protein, fibrinogen, glycated hemoglobin, total cholesterol, triglycerides, glomerular filtration rate, albumin, gamma glutamyltransferase, thyrotropin, and insulin-like growth factor-I.

**Figure 2:**
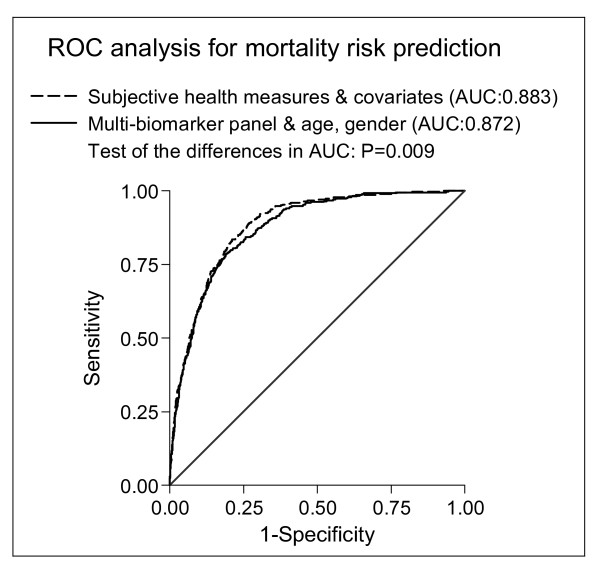
**Receiver-operating characteristic (ROC) curves for mortality risk predicted by subjective health measures and covariates vs.multi-biomarkerpanel**. The Cox model including subjective health measures and covariates incorporated age, gender, self-rated health, PCS-12, MCS-12, civil status, educational level, occupational status, equalized income, smoking status, alcohol consumption, physical activity, food consumption, waist circumference, previous history of hypertension, myocardial infarction, stroke, and diabetes mellitus vs. a multi-biomarker panel including age, gender, high sensitive C-reactive protein, fibrinogen, glycated hemoglobin, total cholesterol, triglycerides, glomerular filtration rate, albumin, gamma glutamyltransferase, thyrotropin, and insulin-like growth factor-I.

Initially, we compared the discriminatory power between the complete self-reported measures panel (incorporating HRQoL, SRH, socio-demographic, behavioral, and comorbidity measures) and the multi-biomarker panel (age, gender, and all 10 biomarkers). The ROC curves depicted in Figure 2 illustrate the significantly better discriminatory power of the self-reported measures (C-statistic of 0.883) compared to the biomarker panel (0.872). To evaluate the added discriminatory power in multivariable Cox models, we implemented a Cox model only including age and gender, yielding a significantly lower C-statistic of 0.843 (p < 0.001). In order to define a parsimonious adjustment set for each panel, the conducted variable selection procedures identified gender, age, occupational status, educational level, cohabitation, smoking, WC, and history of stroke and diabetes mellitus as relevant socio-demographic and behavioral covariates for mortality risk prediction (model 1); and fibrinogen, HbA1c, albumin, and GGT as the most informative biomarkers (model 2). The calculated C-statistics confirmed the better discriminatory power of model 1 (incorporating only SRH and selected socio-demographic and behavioral covariates, respectively) compared to model 2 (age, gender, and the selected biomarkers), yielding a significantly higher C-statistic of 0.883 vs. 0.873 (p = 0.010). Finally, we combined both reduced models (SRH, selected covariates, and selected biomarkers) and detected the best discriminatory power with a significantly higher C-statistic of 0.887 (p < 0.001) compared to the previously presented separate assessment of model 1 (0.883) or model 2 (0.873). We confirmed this finding with a highly significant IDI, estimated at 1.5% (p < 0.001).

Sensitivity analyses with the inclusion of multiplicative interaction terms did not yield any significant effect modification caused by gender or age (p < 0.05). Furthermore, we found virtually no differences comparing the risk estimates between Cox models with and without time-varying covariates. Stratified analyses revealed that especially middle-aged men (40-59 years) were responsive to the detected associations of low PCS-12 (HR, 3.61; 95% CI, 1.43-9.14) and poor SRH (HR, 4.37; 95% CI, 1.27-15.04) with all-cause mortality. Finally, the additional inclusion of non-response weights altered the revealed estimates only slightly (data not shown).

## Discussion

### Principal findings

The present study investigated the associations between subjective health, multiple biomarkers, and mortality risk; offering three principal findings. First, we found that poor SRH and low PCS-12 scores were significantly associated with increased risk of all-cause mortality, independent of a broad spectrum of standard covariates and multiple biomarkers. Second, we found that a risk assessment using subjective health instruments and standard covariates yielded a better mortality risk prediction compared to that of a multi-biomarker panel. Finally, we were able to show that the most accurate mortality risk prediction was obtained from a combined assessment of subjective health and biomarkers.

### HRQoL & mortality

Our presented risk estimates of the association between low PCS-12 and mortality were similar to those previously reported among Taiwanese community-dwelling elderly [12], but much smaller compared to estimates among American community-dwelling elderly [10]. We were not able to detect any association of MCS-12 with all-cause mortality in the present study. This result is in line with previous studies based on particular disease groups [17,19,20,37-39] or community cohorts [10-12], which similarly found PCS-12 but not MCS-12 associated with mortality after multivariable adjustment. Thus, our findings supports the notion that physical domains of HRQoL measures are be more tightly related to mortality compared to mental domains [14,40,41]. As a potential explanation, it is possible that despite the high concordance between PCS-12/MCS-12 and SF-36 scores [2], particular aspects of mental functioning are not captured, ultimately leading to absent associations. However, it has been shown that the mental health status contributes to the PCS-12 as opposed to MCS-12, most likely due to the strong interrelationship between physical and mental domains of health [42]. Thus, the strength of relationship between mental HRQoL and health outcomes may be diluted due to limitations in the applied MCS-12 metric.

### SRH & mortality

Our observed effect sizes between SRH and mortality reflect fairly well the range found in the literature, although the confidence intervals are somewhat wider [7,43]. Our results are in line with previous investigations suggesting SRH not only as an independent predictor of mortality risk, but also as a stronger predictor than HRQoL [40,44]. Because we and previous studies found the relationship between SRH and mortality to be stronger in men than in women [6,45], and stronger in younger than older individuals [46], we conducted sensitivity analyses incorporating interaction terms for gender and age, but without detecting any significant effect modification.

### Biomarker panel

To the best of our knowledge, this is the first population-based study to systematically assess the impact of a comprehensive multi-biomarker panel on the subjective health-mortality association. Our finding of an association between poor SRH and low PCS-12 scores with increased mortality risk was independent of a broad spectrum of multiple biomarkers from distinct biological pathways. This finding is in line with previous results from a five-year follow-up of 4,065 individuals aged 71 years or older, showing SRH significantly associated with mortality risk after adjustment for various biomarkers including albumin, white blood cell count, hemoglobin, high-density lipoprotein cholesterol, and creatinine [47]. It is important to note that this previous study included biomarkers only for additional adjustment, but not to assess their comparative predictive performance with regard to mortality risk.

When we applied variable selection procedures to answer the question which of the 10 biomarkers were most predictive in terms of mortality risk, we identified fibrinogen, HbA1c, albumin, and GGT as the most predictive biomarkers. These pathophysiological highly plausible mortality risk candidate biomarkers reflect disturbances in hemostasis (fibrinogen), metabolism (HbA1c), kidney disease (albumin), and liver disease (GGT). Their predictive ability has been shown to be similar to the full biomarker panel, suggesting improved risk stratification effectiveness using this parsimonious set of biomarkers. But even more interestingly, the predictive performance of these selected four biomarkers was shown to be nearly as good as a risk assessment based on subjective health instruments and standard covariates. While previous studies accumulated evidence that subjective health is a powerful mortality risk predictor, the present findings add to the existing literature indicating that this selected biomarker set plus information about gender and age do about as well. Finally, we were able to show that the combined assessment of subjective health and biomarkers significantly improved the discriminatory ability of the mortality risk prediction model beyond that of each separate panel. There is only one previous study among US veterans that investigated the predictive power of PCS-12 [43]. Compared to our estimates, they reported a slightly lower C-statistic of 0.73, which could be explained by sampling artifacts as veterans tend to be older with a higher proportion of males than in general populations. However, our results suggest that the physiological effects of subjective health measures are synergistic with those captured by the biomarker panel, whereas a combined assessment was identified as the most sensitive barometer of physiologic states associated with increased mortality risk.

### Strengths and limitations

The strengths of our study include a prospective population-based sample of adults aged 20-79 years, a completed 10-year follow-up period utilizing valid and reliable mortality data based on a national death register, comprehensive subjective health and covariable assessment, as well as a broad multi-biomarker panel. Nonetheless, the present study has several limitations. First, non-response bias may potentially exist in this sample, because non-response is particular relevant for self-report of mental properties. But when we performed sensitivity analyses accounting for non-response bias, we detected only minimal deviations from our main results. Second, this study sampled a healthy adult population, with only 11% of participants deceased over the follow-up period. But although this proportion is lower than in several previous studies, a comprehensive review of single-item measured SRH and mortality concluded that studies with lower than 10% mortality found similar effect sizes as studies with higher mortality rates [7]. Even a study with only 5% decedents demonstrated similar effect sizes for the association between SF-12 derived HRQoL and mortality risk [12]. Third, changes in HRQoL over time have been suggested to be as important as the actual baseline value in terms of mortality risk prediction. Studies in selected populations showed that individuals moving from low to high SF-36 scores (improvement) had similar mortality patterns than those who scored high over time [11,48]. Although this analytical approach would have been a valuable extension of the present study based on baseline measurements, it is yet unclear how a change in HRQoL over time is related to mortality in the general population.

## Conclusions

The present results from a large population-based epidemiological study demonstrate that the association of poor SRH and low PCS-12 with mortality is independent of a broad spectrum of standard covariates and multiple biomarkers from distinct biological pathways. But the key finding of the present study is that a small set of biomarkers conjointly with subjective health instruments and socio-demographic standard measures significantly improved the mortality risk prediction above and beyond a separate assessment.

While the first finding is more confirmative in nature, the latter holds important implications from epidemiological and public health perspectives. Incorporating both subjective health assessment and a small set of biomarkers into routine data collection could be used to monitor population health, especially to identify subpopulations particularly at risk. Doing so, the improved identification of high-risk individuals could increase this efficacy of disease prevention strategies. However, further research must be conducted to elucidate how subjective health and biomarkers are interrelated and interdependent. Carrying the present results forward to cause-specific mortality and morbidity will lead to a better understanding of these relationships.

## Competing interests

The authors declare that they have no competing interests.

## Authors' contributions

RH; JM, TK, and HW contributed to the study design and ideas for the data analysis. HV, MD, MN, and HW organized the sample collection and data preparation. Statistical analyses were performed by RH. RH, YSF, JM, HW, and TK contributed to the interpretation of the results and the discussion. RH drafted the manuscript and wrote the final version together with all other co-authors. All authors read and approved the final manuscript.

## Disclosures

The authors have nothing to disclose.

## Pre-publication history

The pre-publication history for this paper can be accessed here:

http://www.biomedcentral.com/1471-2288/11/103/prepub

## Supplementary Material

Additional file 1 **Adjusted hazard ratios (95% CI) for PCS-12, MCS-12, and self-rated health associated with all-cause mortality**. Complete regression results of all Cox proportional-hazards analyses. Click here for file

## References

[B1] Raczek AE Ware JE Bjorner JB Gandek B Haley SM Aaronson NK Apolone G Bech P Brazier JE Bullinger M Sullivan M Comparison of Rasch and summated rating scales constructed from SF-36 physical functioning items in seven countries: results from the IQOLA Project. International Quality of Life Assessment JClinEpidemiol 1998 51 11 1203 1214 10.1016/s0895-4356(98)00112-7 9817138

[B2] Ware J Kosinski M Keller SD A 12-Item Short-Form Health Survey: construction of scales and preliminary tests of reliability and validity Medical care 1996 34 3 220 233 10.1097/00005650-199603000-00003 8628042

[B3] Ware JE Kosinski M Gandek B Aaronson NK Apolone G Bech P Brazier J Bullinger M Kaasa S Leplege A Prieto L Sullivan M The factor structure of the SF-36 Health Survey in 10 countries: results from the IQOLA Project. International Quality of Life Assessment JClinEpidemiol 1998 51 11 1159 1165 10.1016/s0895-4356(98)00107-3 9817133

[B4] Kontodimopoulos N Pappa E Niakas D Tountas Y Validity of SF-12 summary scores in a Greek general population Health and quality of life outcomes 2007 5 55 10.1186/1477-7525-5-55 17900374 PMC2140054

[B5] Burstrom B Fredlund P Self rated health: Is it as good a predictor of subsequent mortality among adults in lower as well as in higher social classes? JEpidemiolCommunity Health 2001 55 11 836 840 10.1136/jech.55.11.836 PMC1763304 11604441

[B6] Heidrich J Liese AD Lowel H Keil U Self-rated health and its relation to all-cause and cardiovascular mortality in southern Germany. Results from the MONICA Augsburg cohort study 1984-1995 AnnEpidemiol 2002 12 5 338 345 10.1016/s1047-2797(01)00300-3 12062922

[B7] Idler EL Benyamini Y Self-rated health and mortality: a review of twenty-seven community studies JHealth SocBehav 1997 38 1 21 37 9097506

[B8] Singh-Manoux A Gueguen A Martikainen P Ferrie J Marmot M Shipley M Self-rated health and mortality: short- and long-term associations in the Whitehall II study PsychosomMed 2007 69 2 138 143 10.1097/PSY.0b013e318030483a PMC4921122 17289825

[B9] Farkas J Nabb S Zaletel-Kragelj L Cleland JG Lainscak M Self-rated health and mortality in patients with chronic heart failure EurJHeart Fail 2009 11 5 518 524 10.1093/eurjhf/hfp038 19329804

[B10] Dorr DA Jones SS Burns L Donnelly SM Brunker CP Wilcox A Clayton PD Use of health-related, quality-of-life metrics to predict mortality and hospitalizations in community-dwelling seniors JAmGeriatrSoc 2006 54 4 667 673 10.1111/j.1532-5415.2006.00681.x 16686880

[B11] Otero-Rodriguez A Leon-Munoz LM Balboa-Castillo T Banegas JR Rodriguez-Artalejo F Guallar-Castillon P Change in health-related quality of life as a predictor of mortality in the older adults QualLife Res 19 1 15 23 10.1007/s11136-009-9561-4 19946754

[B12] Tsai SY Chi LY Lee CH Chou P Health-related quality of life as a predictor of mortality among community-dwelling older persons EurJEpidemiol 2007 22 1 19 26 10.1007/s10654-006-9092-z 17216549

[B13] Gotay CC Kawamoto CT Bottomley A Efficace F The prognostic significance of patient-reported outcomes in cancer clinical trials JClinOncol 2008 26 8 1355 1363 10.1200/JCO.2007.13.3439 18227528

[B14] Quinten C Coens C Mauer M Comte S Sprangers MA Cleeland C Osoba D Bjordal K Bottomley A Baseline quality of life as a prognostic indicator of survival: a meta-analysis of individual patient data from EORTC clinical trials Lancet Oncol 2009 10 9 865 871 10.1016/S1470-2045(09)70200-1 19695956

[B15] McEwen LN Kim C Haan MN Ghosh D Lantz PM Thompson TJ Herman WH Are health-related quality-of-life and self-rated health associated with mortality? Insights from Translating Research Into Action for Diabetes (TRIAD) PrimCare Diabetes 2009 3 1 37 42 10.1016/j.pcd.2009.01.001 PMC4138696 19269911

[B16] Faller H Stork S Schowalter M Steinbuchel T Wollner V Ertl G Angermann CE Is health-related quality of life an independent predictor of survival in patients with chronic heart failure? JPsychosomRes 2007 63 5 533 538 10.1016/j.jpsychores.2007.06.026 17980227

[B17] Rodriguez-Artalejo F Guallar-Castillon P Pascual CR Otero CM Montes AO Garcia AN Conthe P Chiva MO Banegas JR Herrera MC Health-related quality of life as a predictor of hospital readmission and death among patients with heart failure ArchInternMed 2005 165 11 1274 1279 10.1001/archinte.165.11.1274 15956007

[B18] Budweiser S Hitzl AP Jorres RA Schmidbauer K Heinemann F Pfeifer M Health-related quality of life and long-term prognosis in chronic hypercapnic respiratory failure: a prospective survival analysis RespirRes 2007 8 92 10.1186/1465-9921-8-92 PMC2222604 18086309

[B19] Sprenkle MD Niewoehner DE Nelson DB Nichol KL The Veterans Short Form 36 questionnaire is predictive of mortality and health-care utilization in a population of veterans with a self-reported diagnosis of asthma or COPD Chest 2004 126 1 81 89 10.1378/chest.126.1.81 15249446

[B20] Hayashino Y Fukuhara S Akiba T Akizawa T Asano Y Saito S Kurokawa K Low health-related quality of life is associated with all-cause mortality in patients with diabetes on haemodialysis: the Japan Dialysis Outcomes and Practice Pattern Study DiabetMed 2009 26 9 921 927 10.1111/j.1464-5491.2009.02800.x 19719714

[B21] Cunningham WE Crystal S Bozzette S Hays RD The association of health-related quality of life with survival among persons with HIV infection in the United States JGenInternMed 2005 20 1 21 27 10.1111/j.1525-1497.2005.30402.x PMC1490035 15693923

[B22] Salomaa V Havulinna A Saarela O Zeller T Jousilahti P Jula A Muenzel T Aromaa A Evans A Kuulasmaa K Blankenberg S Thirty-one novel biomarkers as predictors for clinically incident diabetes PloS one 5 4 e10100 10.1371/journal.pone.0010100 PMC2852424 20396381

[B23] Zethelius B Berglund L Sundstrom J Ingelsson E Basu S Larsson A Venge P Arnlov J Use of multiple biomarkers to improve the prediction of death from cardiovascular causes The New England journal of medicine 2008 358 20 2107 2116 10.1056/NEJMoa0707064 18480203

[B24] Ingelsson E Pencina MJ Tofler GH Benjamin EJ Lanier KJ Jacques PF Fox CS Meigs JB Levy D Larson MG Selhub J D'Agostino RB Wang TJ Vasan RS Multimarker approach to evaluate the incidence of the metabolic syndrome and longitudinal changes in metabolic risk factors: the Framingham Offspring Study Circulation 2007 116 9 984 992 10.1161/CIRCULATIONAHA.107.708537 17698726

[B25] Haring R Alte D Volzke H Sauer S Wallaschofski H John U Schmidt CO Extended recruitment efforts minimize attrition but not necessarily bias J Clin Epidemiol 2009 62 3 252 260 10.1016/j.jclinepi.2008.06.010 18834716

[B26] Volzke H Alte D Schmidt CO Radke D Lorbeer R Friedrich N Aumann N Lau K Piontek M Born G Havemann C Ittermann T Schipf S Haring R Baumeister SE Wallaschofski H Nauck M Frick S Arnold A Junger M Mayerle J Kraft M Lerch MM Dorr M Reffelmann T Empen K Felix SB Obst A Koch B Glaser S Cohort profile: the study of health in pomerania International journal of epidemiology 2011 40 2 294 307 10.1093/ije/dyp394 20167617

[B27] Alte D Luedemann J Rose HJ John U Laboratory markers carbohydrate-deficient transferrin, gamma-glutamyltransferase, and mean corpuscular volume are not useful as screening tools for high-risk drinking in the general population: results from the Study of Health in Pomerania (SHIP) Alcoholism, clinical and experimental research 2004 28 6 931 940 10.1097/01.ALC.0000128383.34605.16 15201636

[B28] Winkler G Doring A Validation of a short qualitative food frequency list used in several German large scale surveys Zeitschrift fur Ernahrungswissenschaft 1998 37 3 234 241 9800314 10.1007/pl00007377

[B29] Kawachi I Kennedy BP The relationship of income inequality to mortality: does the choice of indicator matter? Social science & medicine (1982) 1997 45 7 1121 1127 10.1016/S0277-9536(97)00044-0 9257403

[B30] Levey AS Bosch JP Lewis JB Greene T Rogers N Roth D A more accurate method to estimate glomerular filtration rate from serum creatinine: a new prediction equation. Modification of Diet in Renal Disease Study Group Ann Intern Med 1999 130 6 461 470 10075613 10.7326/0003-4819-130-6-199903160-00002

[B31] Cook NR Use and misuse of the receiver operating characteristic curve in risk prediction Circulation 2007 115 7 928 935 10.1161/CIRCULATIONAHA.106.672402 17309939

[B32] Pencina MJ D'Agostino RB D'Agostino RB Vasan RS Evaluating the added predictive ability of a new marker: from area under the ROC curve to reclassification and beyond Stat Med 2008 27 2 157 172 discussion 207-112 10.1002/sim.2929 17569110

[B33] Afifi AA Kotlerman JB Ettner SL Cowan M Methods for improving regression analysis for skewed continuous or counted responses Annual review of public health 2007 28 95 111 10.1146/annurev.publhealth.28.082206.094100 17112339

[B34] Donders AR van der Heijden GJ Stijnen T Moons KG Review: a gentle introduction to imputation of missing values Journal of clinical epidemiology 2006 59 10 1087 1091 10.1016/j.jclinepi.2006.01.014 16980149

[B35] Van Buuren S Outshoorn K Flexible multivariate imputation by mice Technical report 1999 Leiden: TNO prevention and Health

[B36] von Elm E Altman DG Egger M Pocock SJ Gotzsche PC Vandenbroucke JP The Strengthening the Reporting of Observational Studies in Epidemiology (STROBE) statement: guidelines for reporting observational studies Lancet 2007 370 9596 1453 1457 10.1016/S0140-6736(07)61602-X 18064739

[B37] Domingo-Salvany A Lamarca R Ferrer M Garcia-Aymerich J Alonso J Felez M Khalaf A Marrades RM Monso E Serra-Batlles J Anto JM Health-related quality of life and mortality in male patients with chronic obstructive pulmonary disease AmJRespirCrit Care Med 2002 166 5 680 685 10.1164/rccm.2112043 12204865

[B38] Mapes DL Lopes AA Satayathum S McCullough KP Goodkin DA Locatelli F Fukuhara S Young EW Kurokawa K Saito A Bommer J Wolfe RA Held PJ Port FK Health-related quality of life as a predictor of mortality and hospitalization: the Dialysis Outcomes and Practice Patterns Study (DOPPS) Kidney Int 2003 64 1 339 349 10.1046/j.1523-1755.2003.00072.x 12787427

[B39] Rumsfeld JS MaWhinney S McCarthy M Jr Shroyer AL VillaNueva CB O'Brien M Moritz TE Henderson WG Grover FL Sethi GK Hammermeister KE Health-related quality of life as a predictor of mortality following coronary artery bypass graft surgery. Participants of the Department of Veterans Affairs Cooperative Study Group on Processes, Structures, and Outcomes of Care in Cardiac Surgery JAMA 1999 281 14 1298 1303 10.1001/jama.281.14.1298 10208145

[B40] Dominick KL Ahern FM Gold CH Heller DA Relationship of health-related quality of life to health care utilization and mortality among older adults Aging ClinExpRes 2002 14 6 499 508 10.1007/BF03327351 12674491

[B41] Lee Y The predictive value of self assessed general, physical, and mental health on functional decline and mortality in older adults JEpidemiolCommunity Health 2000 54 2 123 129 10.1136/jech.54.2.123 PMC1731623 10715745

[B42] Simon GE Revicki DA Grothaus L Vonkorff M SF-36 summary scores: are physical and mental health truly distinct? MedCare 1998 36 4 567 572 10.1097/00005650-199804000-00012 9544596

[B43] DeSalvo KB Fan VS McDonell MB Fihn SD Predicting mortality and healthcare utilization with a single question Health ServRes 2005 40 4 1234 1246 10.1111/j.1475-6773.2005.00404.x PMC1361190 16033502

[B44] Kaplan MS Berthelot JM Feeny D McFarland BH Khan S Orpana H The predictive validity of health-related quality of life measures: mortality in a longitudinal population-based study QualLife Res 2007 16 9 1539 1546 10.1007/s11136-007-9256-7 17899447

[B45] Idler EL Kasl SV Lemke JH Self-evaluated health and mortality among the elderly in New Haven, Connecticut, and Iowa and Washington counties, Iowa, 1982-1986 AmJEpidemiol 1990 131 1 91 103 10.1093/oxfordjournals.aje.a115489 2293757

[B46] Strawbridge WJ Wallhagen MI Self-rated health and mortality over three decades - Results from a time-dependent covariate analysis Research on Aging 1999 21 3 402 416 10.1177/0164027599213003

[B47] Jylha M Volpato S Guralnik JM Self-rated health showed a graded association with frequently used biomarkers in a large population sample J Clin Epidemiol 2006 59 5 465 471 10.1016/j.jclinepi.2005.12.004 16632134

[B48] Kroenke CH Kubzansky LD Adler N Kawachi I Prospective change in health-related quality of life and subsequent mortality among middle-aged and older women AmJPublic Health 2008 98 11 2085 2091 10.2105/AJPH.2007.114041 PMC2636439 18511734

