# Supplementary material for: Self-perceived quality of life predicts mortality risk better than a multi-biomarker panel, but the combination of both does best
Source: BMC Med Res Methodol. 2011 Jul 12;11:103. doi: 10.1186/1471-2288-11-103 (PMC3152941; doi:10.1186/1471-2288-11-103)
Supplement: Additional file 1 — Adjusted hazard ratios (95% CI) for PCS-12, MCS-12, and self-rated health associated with all-cause mortality. Complete regression results of all Cox proportional-hazards analyses. [file 1471-2288-11-103-S1.PDF]

Supplemental Table 1A: Adjusted hazard ratios (HR, 95% CI) for quartiles of PCS-12 associated with all-cause mortality.

	PCS-12					
Q1	1.69 (1.27, 2.26) *	1.75 (1.31, 2.33) *	1.63 (1.22, 2.17) *	1.78 (1.33, 2.40) *	1.60 (1.19, 2.14) *	1.64 (1.19, 2.27) *
Q2	1.10 (0.80, 1.52)	1.12 (0.81, 1.55)	1.08 (0.78, 1.50)	1.13 (0.81, 1.58)	1.08 (0.77, 1.49)	1.08 (0.75, 1.55)
Q3	1.10 (0.79, 1.53)	1.12 (0.81, 1.55)	1.03 (0.74, 1.44)	1.08 (0.77, 1.52)	1.03 (0.74, 1.45)	1.08 (0.75, 1.56)
Q4 (Ref.)	1.00 (Ref.)	1.00 (Ref.)	1.00 (Ref.)	1.00 (Ref.)	1.00 (Ref.)	1.00 (Ref.)
Age, years	1.10 (1.09, 1.11) *	1.10 (1.09, 1.11) *	1.08 (0.07, 1.09) *	1.11 (1.10, 1.12) *	1.10 (1.09, 1.11) *	1.10 (1.08, 1.11) *
Sex (women)		0.48 (0.40, 0.59) *	0.38 (0.31, 0.48) *	0.53 (0.41, 0.67) *	0.49 (0.40, 0.60) *	0.49 (0.39, 0.62) *
Occupational status (four categories)			1.31 (1.10, 1.56) *			
Educational level (three categories)			0.79 (0.67, 0.93) *			
Civil status (cohabiting),			1.57 (0.26, 1.96) *			
Household income, €			1.00 (0.99, 1.01)			
Smoking status (Ref. “Never”)				1.00 (Ref.)		
Former				0.83 (0.65, 1.06)		
Current				2.16 (1.63, 2.84) *		
Riskful alcohol consumption (yes vs. no)				1.21 (0.90, 1.63)		
Physical activity (active)				0.75 (0.60, 0.94) *		
Food consumption (Ref. “Unfavorable”)				1.00 (Ref.)		
Regular				1.07 (0.85, 1.34)		
Optimal				0.92 (0.72, 1.16)		
Waist circumference, cm				1.01 (1.00, 1.02) *		
Myocardial infarction (yes vs. no)					1.06 (0.76, 1.46)	
Stroke (yes vs. no)					0.58 (0.41, 0.83) *	
Diabetes mellitus (yes vs. no)					1.57 (1.23, 1.99) *	
Hypertension (yes vs. no)					1.07 (0.88, 1.30)	
hs-C-reactive protein, mg/l						1.00 (0.99, 1.01)
Fibrinogen, g/l						1.34 (1.15, 1.56) *
Glycated hemoglobin, %						1.08 (0.99, 1.19)
Total cholesterol, mmol/l						1.01 (0.92, 1.11)
Triglycerides, mmol/l						1.04 (0.98, 1.11)
Albumin (urine), mg/l						1.002 (1.001, 1.003) *
Gamma glutamyltransferase						1.10 (1.06, 1.15) *
Glomerular filtration rate, ml/min						1.00 (0.99, 1.01)
Thyrotropin, mU/l						1.01 (0.96, 1.05)
Insulin-like growth factor-I, ng/ml						1.00 (0.99, 1.01)

* $p<0.05$; HR, hazard ratio; 95% CI, 95% confidence interval; PCS-12, Physical Component Summary.

Supplemental Table 1B: Adjusted hazard ratios (HR, 95% CI) for quartiles of MCS-12 associated with all-cause mortality.

	MCS-12					
Q1	0.87 (0.68, 1.12)	0.97 (0.75, 1.24)	0.98 (0.76, 1.26)	1.06 (0.82, 1.37)	0.96 (0.74, 1.23)	0.97 (0.74, 1.28)
Q2	0.87 (0.67, 1.12)	0.91 (0.70, 1.17)	0.98 (0.75, 1.26)	0.95 (0.73, 1.23)	0.92 (0.71, 1.19)	0.93 (0.70, 1.23)
Q3	0.81 (0.63, 1.04)	0.84 (0.65, 1.08)	0.87 (0.68, 1.12)	0.88 (0.68, 1.13)	0.87 (0.68, 1.12)	0.85 (0.65, 1.12)
Q4 (Ref.)	1.00 (Ref.)	1.00 (Ref.)	1.00 (Ref.)	1.00 (Ref.)	1.00 (Ref.)	1.00 (Ref.)
Age, years	1.11 (1.10, 1.12) *	1.10 (1.09, 1.11) *	1.08 (0.07, 1.10) *	1.11 (1.10, 1.13) *	1.10 (1.09, 1.11) *	1.10 (1.09, 1.11) *
Sex (women)		0.49 (0.40, 0.60) *	0.39 (0.31, 0.48) *	0.56 (0.44, 0.72) *	0.50 (0.41, 0.62) *	0.51 (0.40, 0.64) *
Occupational status (four categories)			1.32 (1.11, 1.57) *			
Educational level (three categories)			0.78 (0.66, 0.92) *			
Civil status (cohabiting),			1.59 (0.27, 1.98) *			
Household income, €			1.00 (0.99, 1.01)			
Smoking status (Ref. “Never”)				1.00 (Ref.)		
Former				0.87 (0.68, 1.11)		
Current				2.19 (1.66, 2.89) *		
Riskful alcohol consumption (yes vs. no)				1.13 (0.84, 1.52)		
Physical activity (active)				0.74 (0.59, 0.92) *		
Food consumption (Ref. “Unfavorable”)				1.00 (Ref.)		
Regular				1.08 (0.86, 1.35)		
Optimal				0.93 (0.73, 1.18)		
Waist circumference, cm				1.01 (1.00, 1.02) *		
Myocardial infarction (yes vs. no)					0.96 (0.70, 1.33)	
Stroke (yes vs. no)					0.57 (0.40, 0.81) *	
Diabetes mellitus (yes vs. no)					1.68 (1.33, 2.14) *	
Hypertension (yes vs. no)					1.08 (0.89, 1.31)	
hs-C-reactive protein, mg/l						1.00 (0.99, 1.01)
Fibrinogen, g/l						1.36 (1.16, 1.58) *
Glycated hemoglobin, %						1.10 (1.01, 1.21) *
Total cholesterol, mmol/l						1.00 (0.91, 1.09)
Triglycerides, mmol/l						1.05 (0.98, 1.12)
Albumin (urine), mg/l						1.002 (1.001, 1.003) *
Gamma glutamyltransferase						1.11 (1.06, 1.15) *
Glomerular filtration rate, ml/min						1.00 (0.99, 1.01)
Thyrotropin, mU/l						1.01 (0.96, 1.05)
Insulin-like growth factor-I, ng/ml						1.00 (0.99, 1.01)

* p<0.05; HR, hazard ratio; 95% CI, 95% confidence interval; MCS-12, Mental Component Summary.

Supplemental Table 1C: Adjusted hazard ratios (HR, 95% CI) for self-rated health associated with all-cause mortality.

	**Self-rated health**					
“poor” & “very poor”	1.93 (1.25; 2.98) *	2.07 (1.34, 3.20) *	1.88 (1.21, 2.90) *	2.00 (1.28, 3.12) *	1.87 (1.20, 2.91) *	1.63 (1.02, 2.62) *
“fair”	1.22 (0.80; 1.86)	1.23 (0.81, 1.88)	1.17 (0.76, 1.78)	1.16 (0.75, 1.80)	1.18 (0.77, 1.81)	1.11 (0.70, 1.75)
“very good” or “good” (Ref.)	1.00 (Ref.)	1.00 (Ref.)	1.00 (Ref.)	1.00 (Ref.)	1.00 (Ref.)	1.00 (Ref.)
Age, years	1.10 (1.09, 1.11) *	1.10 (1.09, 1.11) *	1.08 (0.07, 1.09) *	1.11 (1.10, 1.12) *	1.10 (1.09, 1.11) *	1.10 (1.09, 1.11) *
Sex (women)		0.47 (0.39, 0.58) *	0.37 (0.30, 0.47) *	0.53 (0.42, 0.67) *	0.48 (0.39, 0.59) *	0.49 (0.39, 0.62) *
Occupational status (four categories)			1.29 (1.09, 1.54) *			
Educational level (three categories)			0.79 (0.67, 0.93) *			
Civil status (cohabiting),			1.57 (0.26, 1.96) *			
Household income, €			1.00 (0.99, 1.01)			
Smoking status (Ref. “Never”)				1.00 (Ref.)		
Former				0.85 (0.67, 1.09)		
Current				2.23 (1.69, 2.95) *		
Riskful alcohol consumption (yes vs. no)				1.14 (0.85, 1.53)		
Physical activity (active)				0.77 (0.61, 0.96) *		
Food consumption (Ref. “Unfavorable”)				1.00 (Ref.)		
Regular				1.11 (0.89, 1.39)		
Optimal				0.96 (0.75, 1.21)		
Waist circumference, cm				1.01 (1.00, 1.02) *		
Myocardial infarction (yes vs. no)					1.08 (0.78, 1.49)	
Stroke (yes vs. no)					0.59 (0.42, 0.85) *	
Diabetes mellitus (yes vs. no)					1.54 (1.21, 1.96) *	
Hypertension (yes vs. no)					1.04 (0.85, 1.26)	
hs-C-reactive protein, mg/l						1.00 (0.99, 1.01)
Fibrinogen, g/l						1.35 (1.16, 1.57) *
Glycated hemoglobin, %						1.09 (0.99, 1.20)
Total cholesterol, mmol/l						1.00 (0.91, 1.09)
Triglycerides, mmol/l						1.05 (0.98, 1.12)
Albumin (urine), mg/l						1.002 (1.001, 1.003) *
Gamma glutamyltransferase						1.10 (1.06, 1.15) *
Glomerular filtration rate, ml/min						1.00 (0.99, 1.01)
Thyrotropin, mU/l						1.01 (0.97, 1.05)
Insulin-like growth factor-I, ng/ml						1.00 (0.99, 1.01)

* $p<0.05$; HR, hazard ratio; 95% CI, 95% confidence interval.
